# The Histone H3K27me3 Demethylases KDM6A/B Resist Anoikis and Transcriptionally Regulate Stemness-Related Genes

**DOI:** 10.3389/fcell.2022.780176

**Published:** 2022-02-02

**Authors:** Mohammed Razeeth Shait Mohammed, Mazin Zamzami, Hani Choudhry, Firoz Ahmed, Bushra Ateeq, Mohammad Imran Khan

**Affiliations:** ^1^ Department of Biochemistry, Faculty of Science, King Abdulaziz University, Jeddah, Saudi Arabia; ^2^ Centre of Artificial Intelligence in Precision Medicines, King Abdulaziz University, Jeddah, Saudi Arabia; ^3^ Department of Biochemistry, College of Science, University of Jeddah, Jeddah, Saudi Arabia; ^4^ University of Jeddah Centre for Scientific and Medical Research (UJ-`CSMR), University of Jeddah, Jeddah, Saudi Arabia; ^5^ Molecular Oncology Lab, Department of Biological Sciences and Bioengineering, Indian Institute of Technology-Kanpur (IIT-K), Kanpur, India

**Keywords:** anoikis, histone demethylases, *CD44*, *SOX2*, HIF1α

## Abstract

Epithelial cancer cells that lose attachment from the extracellular matrix (ECM) to seed in a distant organ often undergo anoikis’s specialized form of apoptosis. Recently, KDM3A (H3K9 demethylase) has been identified as a critical effector of anoikis in cancer cells. However, whether other histone demethylases are involved in promoting or resisting anoikis remains elusive. We screened the major histone demethylases and found that both H3K27 histone demethylases, namely, KDM6A/B were highly expressed during ECM detachment. Inhibition of the KDM6A/B activity by using a specific inhibitor results in reduced sphere formation capacity and increased apoptosis. Knockout of KDM6B leads to the loss of stem cell properties in solitary cells. Furthermore, we found that KDM6B maintains stemness by transcriptionally regulating the expression of stemness genes *SOX2*, *SOX9*, and *CD44* in detached cells. KDM6B occupies the promoter region of both *SOX2* and *CD44* to regulate their expression epigenetically. We also noticed an increased occupancy of the HIF1α promoter by KDM6B, suggesting its regulatory role in maintaining hypoxia in detached cancer cells. This observation was further strengthened as we found a significant positive association in the expression of both KDM6B and HIF1α in various cancer types. Overall, our results reveal a novel transcriptional program that regulates resistance against anoikis and maintains stemness-like properties.

## Introduction

Normal epithelial cells are non-tumorigenic and are anchorage-dependent, i.e., attach well with the matrix for obtaining nutrition and physiological cues. However, tumorigenic epithelial cells require detachment from the matrix and move to different body sites to initiate metastasis (Simpson et al., 2008; Chaffer and Weinberg, 2011; Buchheit et al., 2014). Several studies described that loss of matrix detachment leads to massive changes throughout both molecular and cellular levels. These modifications are attributed to matrix-detached cells to become anoikis-resistant (Paoli et al., 2013; Buchheit et al., 2014). Matrix-detached cells overcome the anoikis condition through several pathways; one of them is related to the epithelial–mesenchymal transition of ECM-detached epithelial cells (Polyak and Weinberg, 2009; Kim et al., 20122012).

ECM-detached cells overcome the anoikis condition through several pathways. One of them is related to the EMT (epithelial–mesenchymal transition) (Heerboth et al., 2015). Circulating cancer cells activate some genes responsible for activating anti-apoptotic and differentiation pathways; it activates the EMT and downregulates the mechanism depending upon the cell to cell attachment and stimulates the cell adhesion molecule and cell displacement. ECM-detached cancer cells undergo diverging epigenetic changes (Heerboth et al., 2015). ECM-detached cells, to aid in the hypoxic environment of circulating tumor cells, alter cell proliferation, anaerobic glycolysis, and metastasis. HIF-1 alpha (Hypoxia-inducible factor 1 alpha) is the main regulatory factor of hypoxia (Chen and Lou, 2017). Several studies revealed hypoxia modulates the chromatin epigenetic landscape. HIF-1 *α* prompts the expression of several JmjC- Jumonji-domain histone demethylases (KDMs) and histone methylases under hypoxic conditions (Yamane et al., 2006).

The HIF alters the chromatin in different ways by changing the expression level of different KDMs and DNA methyltransferase and increases transcription factors to promote EMTs (Mohyeldin et al., 2010; Tyrakis et al., 2016).

In addition to this, the transcriptomic adjustment of the circulating, matrix-detached tumorigenic cells is also documented due to diverging epigenetic changes at DNA and histone levels (Aceto et al., 2014; Gkountela et al., 2019).

Therefore, in the current study, we aimed to explore the impact of matrix detachment on the epigenome, mainly KDM(s), in various cancer types. Furthermore, we aimed to assess the role of identified KDM(s) in maintaining the stemness and survival of matrix-detached cancer cells.

## Materials and Methods

### Cell Lines and Culture

Various cancer cell lines were maintained in the Dulbecco’s modified Eagle’s medium (DMEM) supplemented with 10% FBS and 1% penicillin–streptomycin (Invitrogen) at 37°C in 5% CO_2_. All the cell lines, namely, HCT116, HeLa, and 22Rʋ1 used in the current study, were obtained from ATCC (United States). The cells were grown to 70–90% confluence, and the media was changed every two days. In addition, the cell lines were routinely checked for any *Mycoplasma* contamination.

### Anoikis (Matrix Detachment) Model

We have followed the well-established method of the cell detachment model in our experiments (Khan et al., 2021). Briefly, cells were grown in an ultra-low attachment plate obtained from Corning (Sigma) in a CO_2_ incubator at 37°C. The assessments of matrix detachment were performed in a cell suspension culture. First, cells were dislodged by simple agitation in the presence of trypsin, followed with washing with PBS, resuspended at 0.5×10^6^ cells/ml in serum-free culture media containing BSA, and finally were cultured in an ultra-low attachment plate at 37°C for various time points, which resulted in the formation of spheroids. The spheroids were treated with either a vehicle control (0.1% DMSO) or with different concentrations of GSK J4 (Abcam-144396, Cambridge, MA United States) for five days. The images were captured by using a Nikon (United States) inverted light microscope. Images were analyzed for size measurement by ImageJ software (https://imagej.net/Invasion_assay).

### Real-Time qPCR Analysis for the mRNA Expression

Briefly, RNA was extracted from all the cell lines at the end of different experimental conditions using the RNAeasy kit (Qiagen) and reverse-transcribed with a high-capacity cDNA reverse transcription kit (applied biosystems). cDNA (1–100 ng) was amplified in triplicates using gene-specific primers (Table 1). Threshold cycle (*C*_*T*_) values obtained from the instrument’s software were used to calculate the respective mRNAs’ fold change. Δ*C*_*T*_ was calculated by subtracting the *C*_*T*_ value of the housekeeping gene from that of the mRNA of interest. ΔΔ*C*_*T*_ for each mRNA was then calculated by subtracting the control’s CT value from the experimental value. The fold change was calculated by formula 2^−ΔΔ*CT*^.

**TABLE 1:** List of primers for real-time PCR.

hJARID1A	5′-TGT​GTT​GAG​CCA​GCG​TAT​GG-3′	5′-CCA​CCC​GGT​TAA​AAG​CAG​ACT-3′
hJARID2	5′-TGT​TCA​CAA​CGG​GCA​TGT​TT-3′	5′-TTG​TGT​TTT​TGA​ACA​GGT​TCC​TTC​T-3′
hKDM3A	5′-GTG​GTT​TTC​AGC​AAC​CGT​TAT​AAA-3′	5′-CAG​TGA​CGG​ATC​AAC​AAT​TTT​CA-3′
hKDM3B	5′-TGC​CCT​TGT​ATC​AGT​CGA​CAG​A-3′	5′GCA​CTA​GGG​TTT​ATG​CTA​GGA​AGC​T-3′
hKDM4A	5′-TGC​AGA​TGT​GAA​TGG​TAC​CCT​CTA-3′	5′-CAC​CAA​GTC​CAG​GAT​TGT​TCT​CA-3′
hKDM4B	5′-GGC​CTC​TTC​ACG​CAG​TAC​AAT​AT-3′	5′-CCA​GTA​TTT​GCG​TTC​AAG​GTC​AT-3′
hKDM4C	5′-GAA​TGC​TGT​CTC​TGC​AAT​TTG​AGA-3′	5′-CAA​CGG​CGC​ACA​TGA​CAT-3′
hKDM6B	5′-CGG​AGA​CAC​GGG​TGA​TGA​TT-3′	5′-CAG​TCC​TTT​CAC​AGC​CAA​TTC​C-3′
hSOX2	5′- TGA​TCA​TGT​CCC​GGA​GGT-3′	5′- CAT​GGG​TTC​GGT​GGT​CAA​G-3′
hSOX9	5′- TCT​ACT​CCA​CCT​TCA​CCT​ACA​T-3′	5′-CTG​TGT​GTA​GAC​GGG​TTG​TT-3′
hCD44	5′- CAG​CAC​TTC​AGG​AGG​TTA​CAT-3′	5′- GTA​GCA​GGG​ATT​CTG​TCT​GTG-3′
LinVEGFA	5′-GCC​TCC​GAA​ACC​ATG​AAC​TTT-3′	5′-CCA​TGA​ACT​TCA​CCA​CTT​CGT-3′
Lin HIF 2α	5′-CTG​AAC​GTC​TCA​AAG​GGC​CA-3′	5′-CCT​TCC​TCC​TCT​CCG​AGC​TA-3′
Lin HIF 1α	5′-ATG​CTT​TAA​CTT​TGC​TGG​CCC-3′	5′-TCT​GTG​TCG​TTG​CTG​CCA​AA-3′
hKDM6A	5′-CAC​AGT​ACC​AGG​CCT​CCT​CAT​T-3′	5′-TCA​CTA​TCT​GAG​TGG​TCT​TTA​TGA​TGA​CT-3′

### Histone Demethylase Activity Assay

Histone H3K27 demethylase KDM6A/B activity was measured by using the Abcam- KDM6A/B activity quantification kit (ab156910). The protocol was performed based on the Kit guidelines. 15 µg of the nuclear extract was used. The nuclear protein was extracted without using detergent. The ECM-detached cells with and without treatment of GSK J4 and ECM-attached cells 2 × 10^6^ were obtained. They were washed with ice-cold PBS three times in 500 g for 10 min in 500 µL of lysis buffer 10 mM HEPES, pH 7.9, with 1.5 mM MgCl_2_ and 10 mM KCl (add 5 µL of 0.1 M DTT and 5 µL of protease inhibitor cocktail) and incubated for 15 min in ice. The supernatant was removed after spinning, and the pellets were resuspended in 200 µL of the lysis buffer, and five gentle strokes were performed using a glass homogenizer. It was allowed to spin for 20 min at 12,000 g. The cytosolic fraction (supernatant) was removed, and 150 µL of nuclear extraction buffer 20 mM HEPES, pH 7.9, with 1.5 mM MgCl_2_, 0.42 M NaCl, 0.2 mM EDTA, and 25% (v/v) glycerol was added with the addition of 1.5 µL of 0.1 M DTT and 1.5 µL of protease inhibitor cocktail. It was incubated in ice for 30 min with a gentle shake every 2 min and allowed to spin at 21,000 g for 10 min to obtain the supernatant (Nuclear protein).

### H3K27 Methyltransferase Activity Assay

The H3K27-specific methyltransferase activity was performed using a commercially available kit from Abcam [H3K27 methylation Assay Kit (Colorimetric) # ab156910]. The assay kit could measure the activity or inhibition of H3K27 mono/di/tri subtypes and required cellular nuclear extracts. Briefly, equal amounts of protein samples were added in each independent experiment; however, the overall protein concentration ranged from 100 to 300 ng H3K27 modifications were calculated according to the manufacturer’s instructions, which also accounts for protein amounts, and the final values for each modification were presented as the percentage over untreated control.

### Protein Extraction and Western Blot Analysis

Various cancer cells (HCT116, HeLa, and 22Rv1) were cultured in a T_75_ flask (1 × 10^6^/flask). After 24 h, plating cells in ultra-low attachment plates were treated with GSK J4 for the indicated dose for five consecutive days. The new treatment was added every 48 h; following completion of treatment, media was aspirated, and cells were washed with cold PBS (pH 7.4) and pelleted in 15-ml falcon tubes. Ice-cold lysis buffer was added to the pellet. The composition of the lysis buffer was 50 mM Tris–HCl, 150 mM NaCl, 1 mM ethylene glycol-bis(aminoethyl ether)-tetraacetic acid, 1 mM ethylenediaminetetraacetic acid, 20 mM NaF, 100 mM Na_3_VO_4_, 0.5% NP-40, 1% Triton X-100, and 1 mM phenylmethylsulfonyl fluoride, pH 7.4 with freshly added protease inhibitor cocktail (Protease Inhibitor Cocktail Set III, Calbiochem, La Jolla, CA). Then, cells were passed through the needle of the syringe to break up the cell aggregates. The lysate was cleared by centrifugation at 14,000 *g* for 30 min at 4 °C, and the supernatant (nuclear lysate) was used or immediately stored at −80°C. For western blotting, 4–12% polyacrylamide gels were used to resolve 30 μg of protein, transferred onto a nitrocellulose membrane, probed with appropriate monoclonal primary antibodies, and detected by chemiluminescence after incubation with specific secondary antibodies (Shait Mohammed et al., 2020).

### Flow Cytometry Analysis

HeLa, HCT116, and 22Rʋ1 cells were grown in an ultra-low attachment plate for six days with and without treatment of GSK J4. Cells were washed two times in ice-cold PBS and resuspended in 100 µL of staining solution (5 µL PE-conjugated anti-CD 70 (BD Bioscience), CD 133 (Miltenyibiotec-130-090-853), and FITC-conjugated anti-CD 105(BD Bioscience- 56143) and anti-CD44(Miltenyibiotec -130-098-210), 1% FBS, in 1X PBS and incubated at room temperature under the dark condition for 2 h. Then, cells were washed three times in a wash buffer (1%FBS in 1X PBS) and then analyzed by using a Guava Easy-Cyte flow cytometer. For all assays, 10,000 cells were taken for measurement and also for analysis. For each plot, 1000 cells were displayed.

### Immunofluorescence

For immunofluorescence assay, cells were grown in ultra-low attachment plates for six days with and without treatment of GSK J4. Then, cells were collected and washed two times with ice-cold PBS, and cells were stained with a staining solution containing (5 μL, CD 133 and FITC anti-CD 105, and anti-CD44) 1% FBS in 1X PBS and incubated at room temperature in the dark for 2 h. Then, cells were washed two times in ice-cold PBS, and cells were analyzed using a Amins image stream flow cytometer.

### Apoptosis Assay

The apoptotic cells were detected by using Annexin V-FITC and propidium iodide. The cells were grown in an ultra-low attachment plate for six days with and without treatment with GSK J4. After treatment, spheroids were harvested and washed with PBS (ice-cold) three times. The spheroids were broken down by multiple pipetting and resuspended in 100 μL 1X binding buffer, 10 μL Annexin V-FITC, and 5 μL *p*I. After incubation for 20 min in RT (dark condition), the cells were analyzed by using The Guava^®^ easy, yet 5 flow cytometer, and the percentage of apoptosis cells was calculated (Alzahrani et al., 2021).

### CHIP PCR

The CHIP experiments were performed by using the Abcam (ab185913) chip kit. The protocol was followed as per standard guidelines of the manual with minor modifications. The spheroids (ECM detached), ECM-attached cells, and ECM-detached cells were harvested and washed with ice with 3 ml PBS. Cells were resuspended in 1% formaldehyde in the DMEM and incubated at RT for 10 min with the minor rocking platform, and 300 μL of 1.25 M glycine was added for crosslinking washed with ice-cold PBS. The pellet was resuspended in 300 μL lysis buffer, and the chromatin was sheared using a water bath sonicator (15 cycles on and 15 cycles off for 20 min). Three micrograms of the sheared chromatin were taken to the well coated with KDM6B antibodies, input (negative control) along with nonimmune IgG and incubated overnight at 4°C. The unbounded chromatins were removed and washed with the wash buffer. DNA was released using the DNA release buffer and incubated at 60°C for 45 min in a water bath; subsequently, the solution was transferred to PCR tubes heated at 95°C for 15 min at a thermocycler. The DNA was purified by using the column provided in the kit. The PCR was performed by using the targeted primers (Table 2) of specific genes.

**TABLE 2:** List of primers for CHIP-q PCR.

Chip-CD44	5′- CTG​GCA​GCC​CCG​ATT​ATT-3′	5- AGC​GAG​CGA​AGG​ACA​CAC-3′
Chip-Sox9	5′- GCT​CTA​AGC​ATT​TCG​TGT​AA-3′	5′- TAC​GAA​ACA​CCT​GAA​GGG-3′
Chip-SOX2	5′- CGA​CAA​CAA​GAG​AAA​CAA​AAC-3′	5′- CCA​GCA​AGG​CCC​GGG​TTA-3′
Chip-HIF1α	5′ GAA​GTT​TAC​AGC​AAC​AGG​AG-3′	5′- TTA​CAA​CGG​GGT​CTT​TCC​TTA​C-3′

### Gene Expression Correlation Analysis

For the correlation analysis of gene expressions among *KDM6B*, *SOX2*, *CD44*, and HIF1α, a gene expression profile was taken from two different studies. 1) A metastatic melanoma sample was taken from Hugo et al., 2016 (PMID: 26997480). The study provided the normalized gene expression data in FPKM values from 27 samples. 2) Metastatic breast cancer data of the Metastatic Breast Cancer Project (www.mbcproject.org). The study provided the normalized gene expression data in RSEM values from 146 samples. The normalized expression values were transformed into log2 (x+0.1), and then, the Pearson correlation coefficient was measured with the ggpubr package.

### Statistical Analysis

Data were analyzed using GraphPad Prism (version 5; GraphPad Software). The two-tailed, unpaired *t-*test was used. Data points in graphs represent mean ± SD, and *p* values <0.05 were considered significant.

### CRISPR Knockout of KDM6B

CRISPR case-based gene knockout experiments, the plasmid-containing CRISPR double nickase was constructed by SANTA CRUZ biotechnology sc-401883-NIC for KDM6B and non-targeted + ve control (sc-437281). For plasmid transfection, 1 × 10^5^ per HeLa cells were plated at a 6-well plate. First, 1 μg plasmid was transfected by using Lipofectamine 3000. After transfection, at 48 h, the cells were collected for extraction of total protein. The knockout efficiency was verified by Western blotting.

## Results

### KDM6A/B Expression and Demethylase Activity Are Increased During Anoikis

**FIGURE 1:**
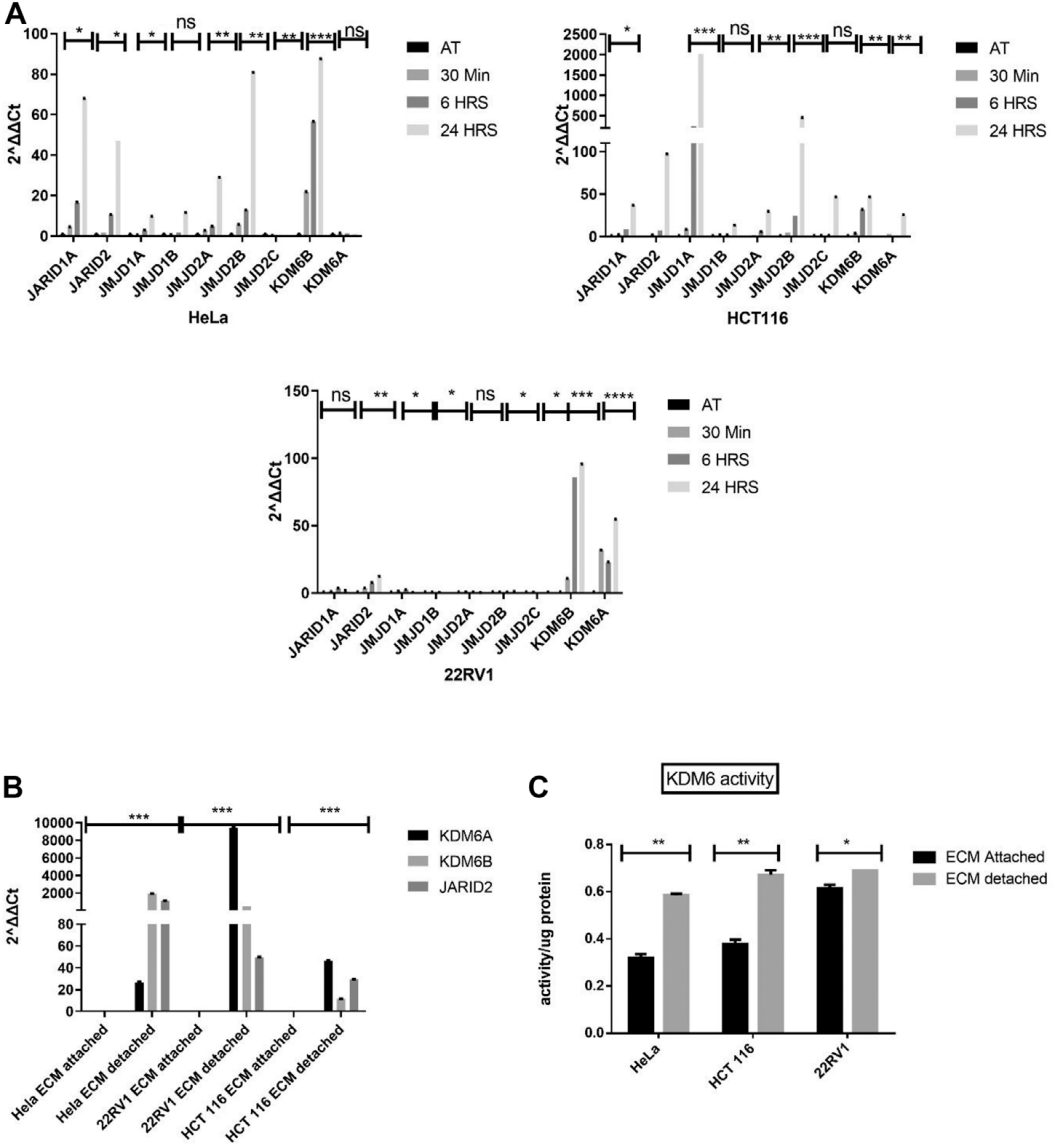
Matrix detachment induces the KDM6B expression and activity. **(A)** Expression of various hypoxia-regulated histone demethylases in HeLa, HCT116, and 22Rv1 cell lines during matrix detachment at shorter time points (30 min, 6, and 24 h). **(B)** JARID2 and KDM6B/A in HeLa, HCT116, and 22Rʋ1 cell lines during matrix detachment for six days. The values were normalized with the housekeeping gene *RPLP0*, and other gene expression values were calculated. **(C)** KDM6 demethylase activity was measured in the nuclear extract of HeLa, HCT116, and 22Rv1 cells grown in matrix-detached conditions. **p.value > 0.001, *p.value > 0.01, ***p.value > 0.0001.

To investigate key histone demethylase, promote or regulate anoikis resistance. We measured the expression of various histone demethylases in detachment conditions of different cancer cell types (HeLa, HCT116, and 22Rv1). Quantitative gene transcript analysis at early time points (from 30 min to 24 h) showed statistically significant induction of various histone demethylases such as JARID1A, JARID2, JMJD1A, and KDM6A/B in all the three cancer cell types grown in detached conditions (Figure 1A). We decided to investigate the above-tested histone demethylases’ expression pattern in long-term detachment conditions (6 days). Only histone demethylase, namely KDM6A/B, stands out as its expression was consistently upregulated in all three cell lines during detached conditions compared with attached (Figure 1B).

Since both KDM6A/B are H3K27 me3 demethylases and were most consistently upregulated at all time points in all cell types, we decided to focus our further work on KDM6A/B. We performed quantitative activity assay for KDM6A/B demethylases and found a statistically significant increase in the KDM6A/B demethylase activity in HeLa, 22Rv1, and HCT116 cell lines in detached conditions when compared with the attached. (Figure 1C). Based on the above data, ECM detachment of cancer induces the expression and activity of KDM6A/B histone demethylases, removing the repressive H3K27me3 mark across the genome, thereby facilitating transcription.

### Targeting KDM6A/B Demethylases Reduces Sphere-Forming Capabilities and Induces Apoptosis of Cancer Cells During Anoikis

**FIGURE 2:**
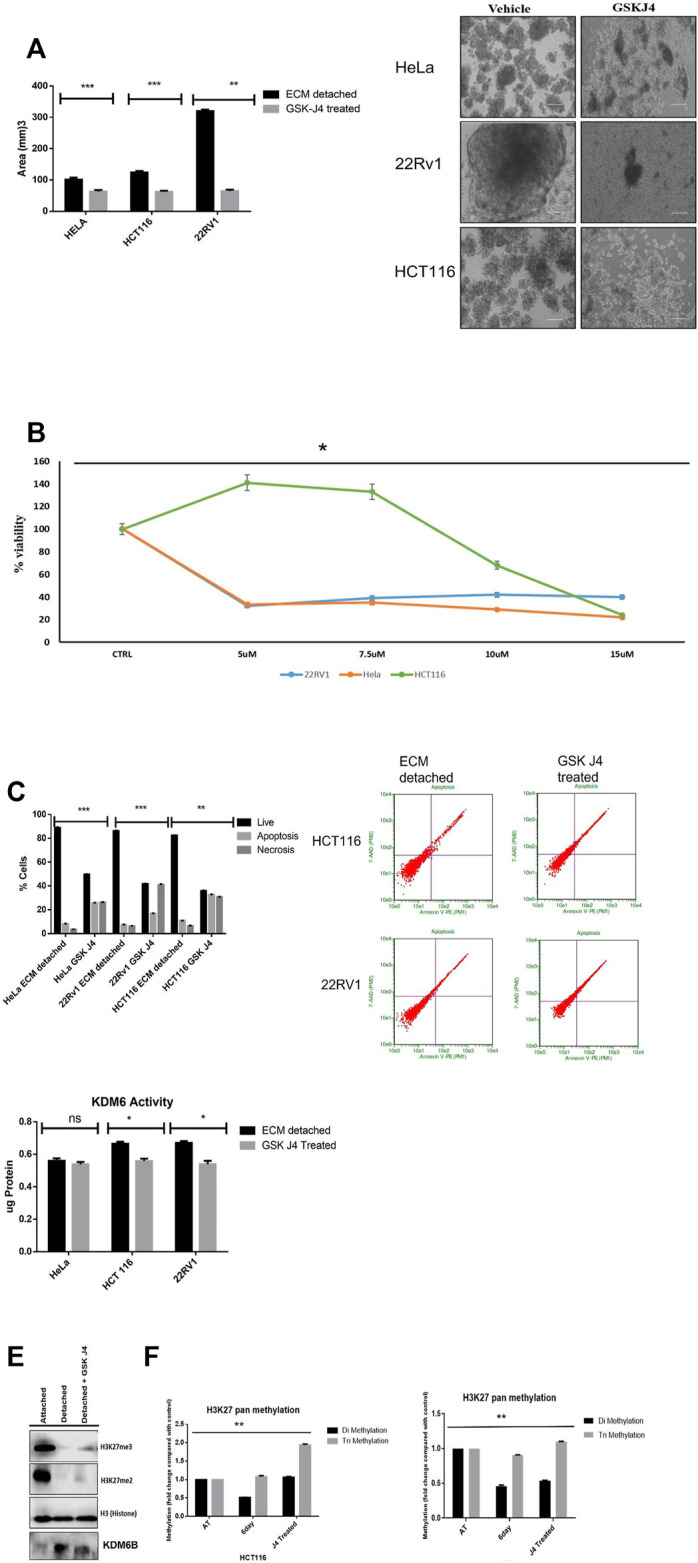
KDM6A/B inhibition reduces the spheroid size and induces apoptosis. **(A)** Sphere formation assay HeLa, HCT116, and 22Rv1 cell lines were cultured in matrix detachment for six days and simultaneously treated either with GSK J4 (5 µM for HeLa and 22Rv1, 10 µM for HCT116) or with vehicle 0.1% DMSO (control). At the end of the treatment schedule, sphere images were captured using a Nikon inverted light microscope, and images were analyzed for size measurement by ImageJ software *N* = 8. Histogram plots were plotted for average size in all conditions. **(B)** HeLa, HCT116, and 22Rv1 cell lines were cultured in matrix detachment for six days and simultaneously treated with different GSK J4 or with vehicle 0.1% DMSO (control). Cell proliferation was measured using WST-1, and IC 50 was calculated. **(C)** Apoptosis assay HeLa, HCT116, and 22Rv1 cell lines were cultured in matrix detachment for six days and simultaneously treated either with GSK J4 (5 µM for HeLa and 22Rv1,10 µM for HCT116) or with vehicle 0.1% DMSO (control). As mentioned above, a similar treatment strategy was used, and apoptosis assay was performed using Annexin V and *p*I. The histogram data were plotted for % cells showing live or apoptotic populations. **(D)** KDM6B/A demethylase activity was measured from the nuclear extract of HeLa, HCT116, and 22Rv1 cell lines and were cultured in matrix detachment for six days and simultaneously treated either with GSK J4 (5 µM for HeLa and 22Rv1,10 µM for HCT116 KDM6B/A) or with vehicle control. The activity was measured using the KDM6 activity assay kit. **(E)** Histone was extracted from ECM-attached and ECM-detached cells. ECM-detached cells treated with GSK-J4 using a histone extraction kit (ab113476) and 10 µg of histone protein loaded for Western blot; Western blot showing GSK J4 treatment for six days significantly induces the expression of H3K27me2/3 in 22RV1 during matrix detachment conditions. **(F)** pan H3k27 me2 and me3 was measured from histone extracted as previous conditions mentioned above. *p*.value **p.value > 0.001, *p.value > 0.01, ***p.value > 0.0001.

Matrix detachment of cancer cells tends to form spheroids. Therefore, to investigate the effect of KDM6A/B inhibition in spheroid formation, we treated spheroids of HeLa, HCT116, and 22Rv1 that formed during matrix detachment with GSK J4 (Heinemann et al., 2014), a highly specific inhibitor of KDM6A/B. Results showed that GSK J4 significantly reduced the size of spheroids of all cancer cell lines tested compared to untreated, respectively (Figure 2A), and IC50 for different dose concentrations was measured before treatment (Figure 2B).

The reduction in the spheroid size is associated with cell death. So we measure the percentage of cell death that might have occurred in ECM-detached cancer cells during GSK J4 treatment. As expected, GSK J4 significantly induces apoptosis in all the ECM-detached cancer cells. Among them, 22Rv1 showed the maximum percentage of apoptotic cells (57.7%) when compared with HeLa (34.4%) and HCT116 (22.8%) (Figure 2C). Overall, we observed that reducing the KDM6A/B activity significantly reduces the sphere-forming capacity and invokes apoptosis in ECM-detached cancer cells.

We observed that GSK J4 treatment significantly reduces the KDM6A/B activity in all cell lines and KDM6B expression (Figures 2D,E), along with GSK J4 treatment which induces global H3K27me2/3 methylation (Figures 2E,F).

### Targeting KDM6A/B Demethylases Reduces the Expression of Stemness-Related Genes During Anoikis

**FIGURE 3:**
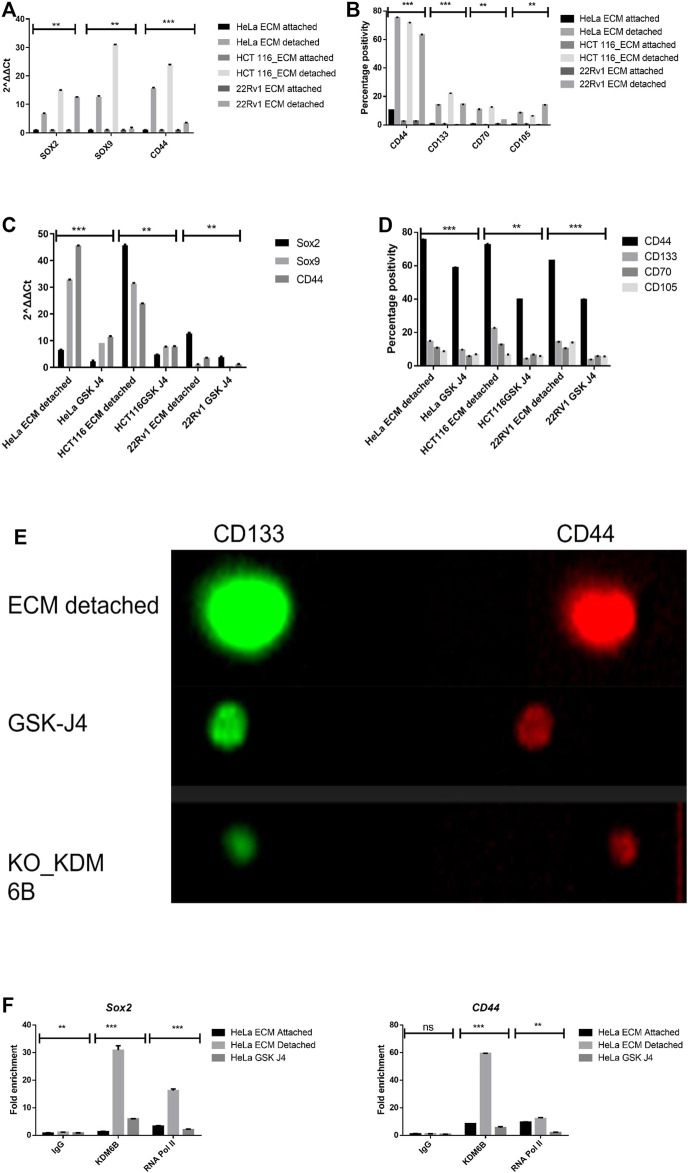
Matrix detachment induces the expression of stemness markers that are repressed by the KDM6B-specific inhibitor. **(A)** mRNA expression of various stemness-related genes (*SOX2*, *SOX9*, and *CD44*) in HeLa, HCT116, and 22Rv1 cell lines during matrix detachment at six days. The values were normalized with the housekeeping gene *RPLP0*, and other gene expression values were calculated. **(B)** Flow cytometry–based expression of various surface markers of stemness in HeLa, HCT116, and 22Rv1 cell lines during matrix detachment for six days. **(C)** Spheroids were treated with GSK-J4 (5 µM for HeLa and 22Rv1,10 µM for HCT116), a specific inhibitor of KDM6 histone demethylases. Moreover, the mRNA expression of various stemness-related genes (*SOX2*, *SOX9*, and *CD44*) in HeLa, HCT116, and 22Rʋ1 cell lines were measured as explained above. **(D)** Spheroids were treated with GSK-J4 (5 µM for HeLa and 22Rv1,10 µM for HCT116). In addition, the flow cytometry–based expression of various surface markers of stemness was measured. **(E)** Immunofluorescence for various stemness surface markers was measured in spheroids and spheroids treated with GSK-J4 in HeLa. **(F)** Enrichment of KDM6B and RNA Pol II on the promoters of *SOX2* and *CD44* genes in the presence and absence of GSK J4 in ECM-detached (spheroids) cells; nonimmune IgG was used as the input control. **p.value > 0.001, *p.value > 0.01, ***p.value > 0.0001.

During matrix detachment, cancer cells often present with induced expression of stemness genes; therefore, we quantified the expression of genes related to stemness such as *SOX2*, *SOX9*, and *CD44* and found significantly increased levels of these stemness-related genes in all matrix-detached cancer cell lines when compared to their attached counterparts (Figure 3A). Furthermore, we further validated the stemness by measuring the surface protein marker of stemness such as CD44, CD133, and mesenchymal markers, namely, CD70 and CD105, by flow cytometry. As expected, we observed statistically significant upregulation of surface stemness and mesenchymal markers in all detached cancer cells compared to attached cells. Thus, our data represent that detachment induces stemness (Figure 3B).

To investigate the role of KDM6A/B demethylases in the transcriptional regulation of stemness genes, we treated all the detached cancer cell types with GSK J4 (a KDM6A/B) for a total period of 5 days after the initial 24 h of anoikis (detachment). Our data showed that GSK J4 treatment significantly reduces the transcript levels of *SOX2*, *SOX9*, and *CD44* genes in detached cancer cells compared to the untreated control (Figure 3C). Next, we assess the impact of GSK J4 treatment on the expression of stemness-related surface proteins by using both flow cytometry and immunofluorescence and found that GSK J4 significantly reduced the expression of *CD44*, *CD133*, *CD70*, and *CD105* in detached cells when compared to untreated (Figures 3D,E; Supplementary Figure S1).

We next aimed to investigate the KDM6B occupancy on stemness genes’ promoters during anoikis conditions. For this, we performed a CHIP-RT PCR assay. As a result, we observed significant enrichment of KDM6B on promoter regions of *SOX2* and *CD44* but not on *SOX9* in detached cancer cells. Furthermore, GSK J4 treatment significantly reduces the occupancy of KDM6B on promoters of both *SOX2* and *CD44*, suggesting the KDM6B-driven regulation of these stemness genes in detached cancer cells (Figure 3F).

### Targeting KDM6A/B Demethylases Reduces the HIF-1α Expression During Anoikis

**FIGURE 4:**
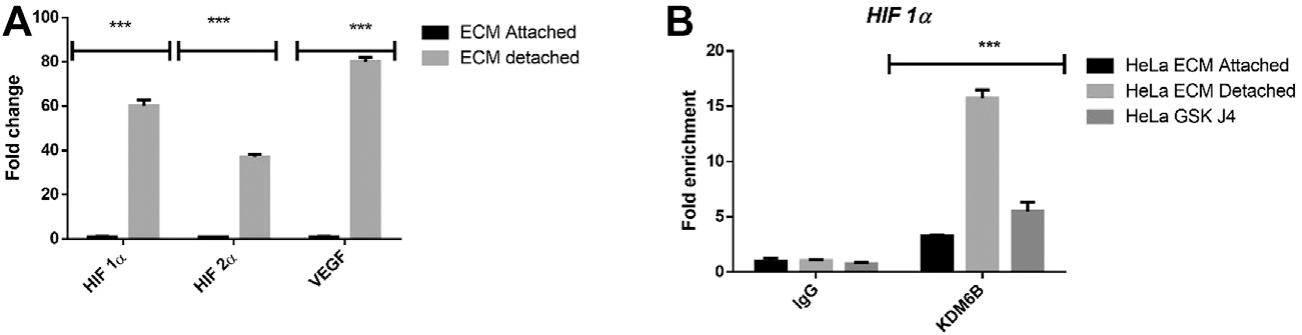
Matrix detachment induces the hypoxia-regulated KDM6B expression and activity. **(A)** mRNA expression of various stemness-related genes HIF1α, HIF2 α, and target gene VEGF in HeLa cell lines during matrix detachment for six days. The values were normalized with the housekeeping gene *RPLP0*, and other gene expression values were calculated. **(B)** Enrichment of KDM6B on the promoters of HIF 1α both in the presence and absence of GSK J4 (5 µM) in HeLa cells during matrix detachment for six days; nonimmune IgG was used as the input control. **p.value > 0.001, *p.value > 0.01, ***p.value > 0.0001.

Hypoxia-driven regulation of the histone demethylase expression and activity is a well-established phenomenon. Recent studies have shown that various histone methyltransferases such as SET7/9, G9a, and GLP can methylate HIF1Α and regulate and repress its expression (Bao et al., 2018). It prompted us to investigate whether induction in the KDM6B activity during ECM detachment might positively regulate the hypoxia-related transcription factors HIF1α and HIF2α expressions and reduce KDM6B activity (using GSKJ4) negatively regulate its expression (Figure 4A). Thus, we investigated whether KDM6B occupies hypoxic transcription factors’ promoters and regulates their expression. Results showed a clear enrichment of KDM6B on the HIF1α promoter and a subsequent reduction in the enrichment by GSK J4, further confirming the transcriptional regulation of HIF1α in matrix-detached conditions (Figure 4B).

### The KDM6B Knockout by CRISPR Reduces the Spheroid Size and Stem Cell Markers

**FIGURE 5:**
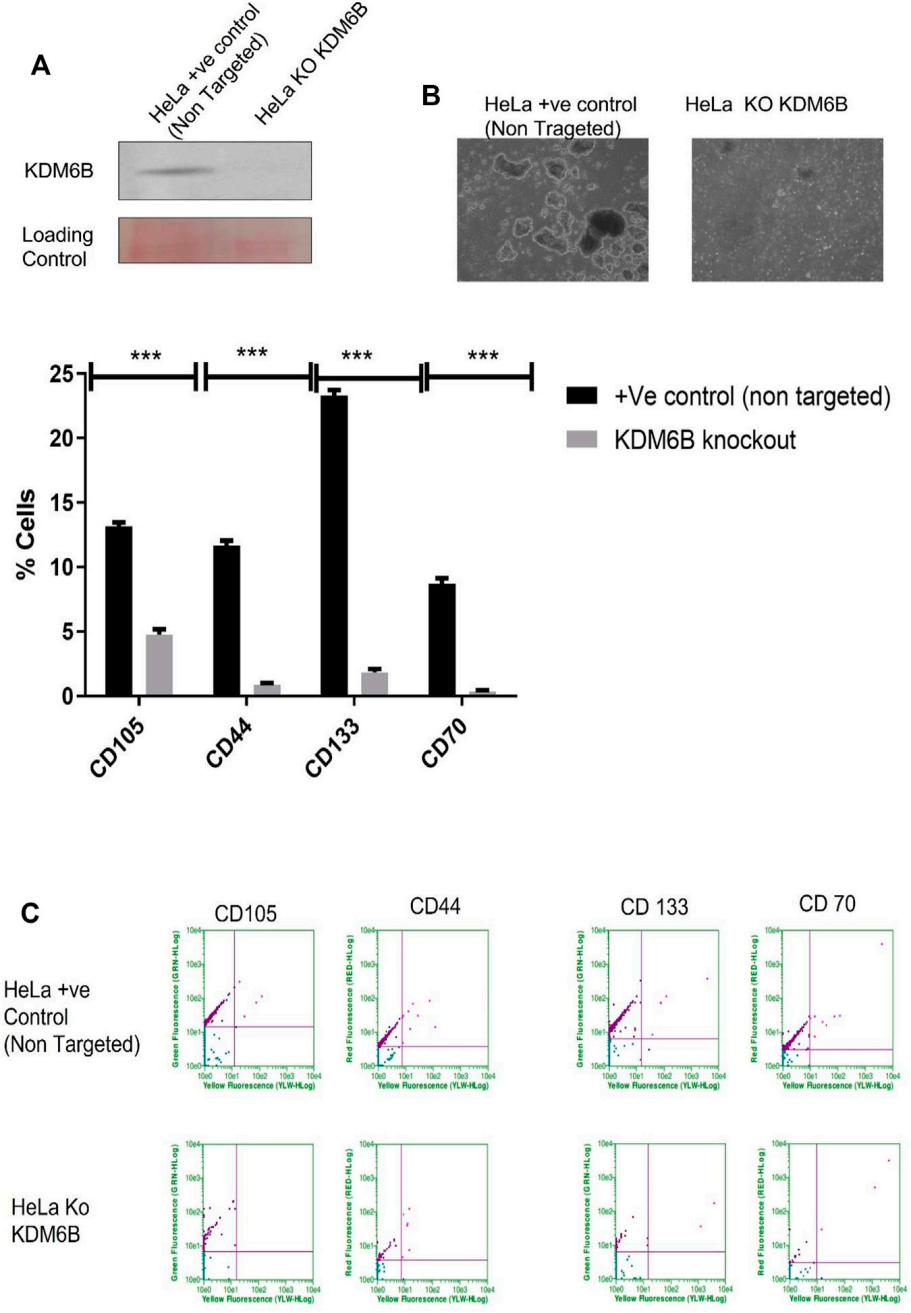
CRISPR knockout KDM6B and reduce stemness in ECM-detached cells. **(A)** Total protein was isolated from +ve control empty CRISPR Cas-transfected cell line and CRISPR KDM6B-knockout cell lines. Western blot was performed as previously mentioned, and the blot was probed with anti-human KDM6B antibodies; the loading control was blot stained with Ponceau staining. **(B)** Sphere formation assay HeLa + ve Control (non-targeted) and HeLa KDM6B-knockout cell lines were cultured in matrix detachment for six days. At the end of the treatment schedule, sphere images were captured by using a Nikon inverted light microscope. **(C)** HeLa + ve Control (non-targeted) and HeLa KDM6B-knockout cell lines were cultured in matrix detachment for six days in the end for time duration. The flow cytometry–based expression of various surface markers of stemness was measured.

To investigate the role of KDM6B in the expression regulation of stem cell markers, we used the CRISPR Double Nickase KDM6B plasmid, which can inhibit the gene expression level of KDM6B. Our results showed that CRISPR, Figure 5A completely removed the protein level of KDM6B in HeLa cells. The spheroid size was decreased (Figure 5B).To examine the KDM6B role in maintaining stem cell markers, we cultured the HeLa KDM6B-knockout cells in the ECM-detached condition. After six days, we examined for stem cell surface markers by flow cytometry. We found that KDM6B knockout significantly reduced the expression of *CD44*, *CD133*, *CD70*, and *CD105* in detached cells when compared to a positive control (Figure 5C).

### Clinical mRNA Expression Association of KDM6B and HIF1α

**FIGURE 6:**
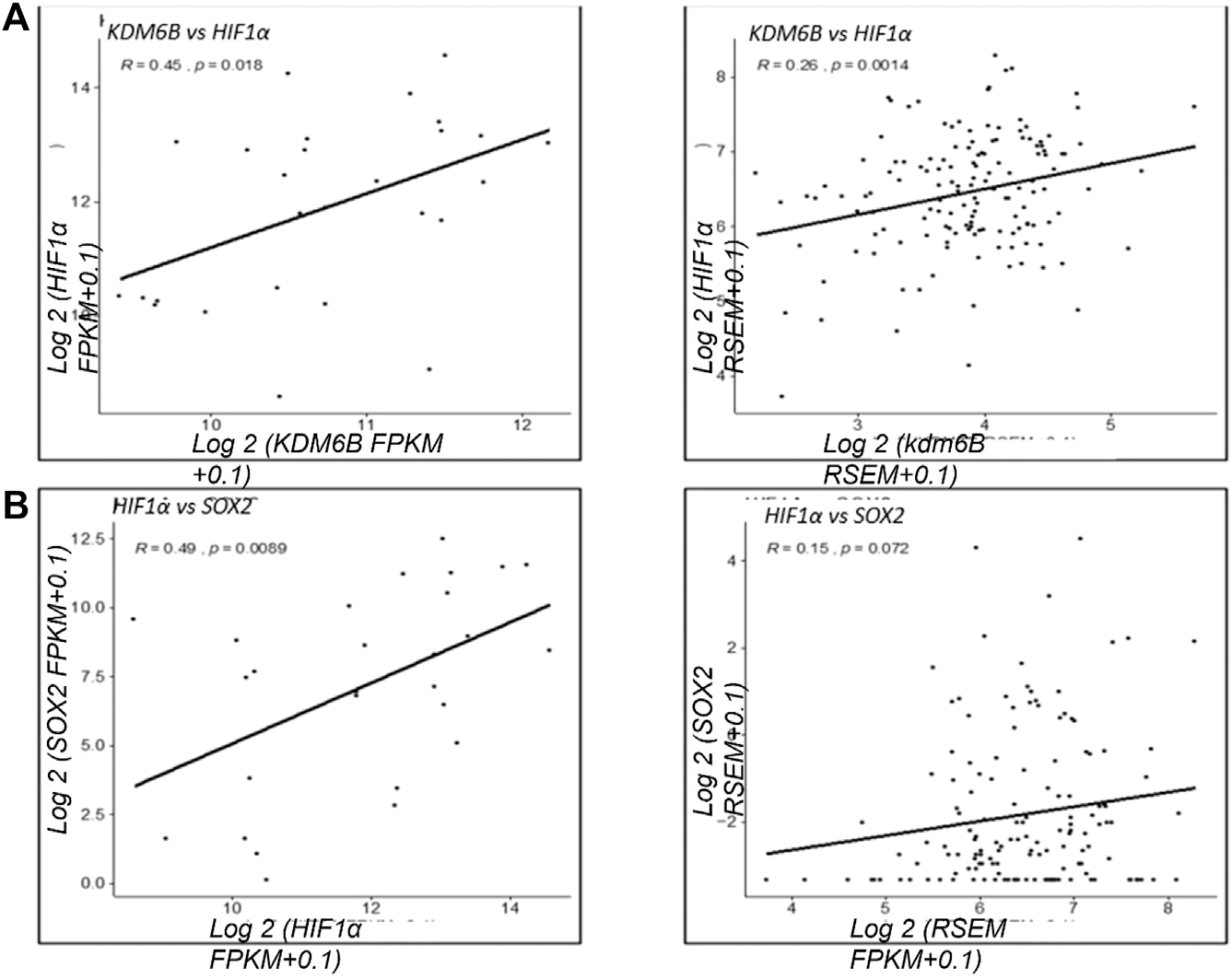
Clinical correlation of KDM6B and HIF 1α in various cancer types. Correlation analysis of the gene expression between KDM6B and HIF 1α from metastatic samples. **(A)** Gene expression in metastatic melanoma samples. **(B)** Gene expression in metastatic breast samples **p.value > 0.001, *p.value > 0.01, ***p.value > 0.0001.

Based on our results that KDM6B transcriptionally regulates the expression of HIF1α, we next investigated the positive association between KDM6B and HIF1α in clinical samples using TCGA datasets. We found a positive association between KDM6B and HIF1α in various cancer types. In metastatic melanoma and breast cancer samples, a significantly high positive correlation was observed between KDM6B and HIF1α mRNA (R = 0.45, *p*-value = 0.018; R = 0.26, *p*-value = 0.0014) (Figures 6A,B). Similarly, we found a significant positive association between HIF1α and *SOX2* (R = 0.49, *p*-value = 0.0089) along with *CD44* and *SOX2* (R = 0.39, *p*-value = 0.045) in melanoma cancers. We also found that metastatic breast samples had a significantly high positive correlation between CD44 and HIF1α (R = 0.24, *p*-value = 0.0031) (Supplementary Figure S2).

## Discussion

Anoikis (matrix detachment) can be regulated by histone demethylases such as KDM3B (Pedanou et al., 2016). However, whether other histone demethylases regulate this process is not well explored yet. In the current work, we found that 1) During anoikis, matrix detachment induces the expression of KDM6A/B, a histone H3K27me2/3 demethylase in all cell types tested. 2) Inhibition of the KDM6A/B activity reduces the expression of stemness-related genes, namely, SOX2 and CD44 and were found to be highly expressed during matrix detachment. 3) Mechanistically, KDM6B occupies the promoter regions of stemness-related genes, thereby regulating their expression. 4) We also noticed that HIF1α was transcriptionally regulated by KDM6B during anoikis conditions. 5) Finally, we observed a positive association between KDM6B, HIF1α, and SOX2 mRNA in various cancer types. Overall, we found that matrix detachment modulates the epigenome during anoikis, inducing KDM6A/B that positively regulates the expression of *SOX2*, *CD44*, and HIF1α to regulate survival and stemness of cancer cells.

KDM6B was shown to regulate epithelial to mesenchymal (EMT) conversion of cancer cells by regulating the expression of the EMT process’s essential transcription factors (Ramadoss et al., 20122012; Liu et al., 2018). Our results suggest KDM6B as a critical regulator of metastasis in different cancer types. Our findings that increased the KDM6B expression and activity are essential for spheroid maintenance during matrix detachment and align well with these previous studies.

Various histone methylases, including KDM6B, regulate normal and cancer stem cells (Jeong et al., 2010; Akiyama et al., 2016; Tang et al., 2016). This study found that blocking the KDM6B activity showed a dramatic reduction in the expression of stemness marker genes, mainly *SOX2*, *SOX9*, and *CD44*, suggesting their epigenetic regulation in matrix-detached conditions. The post-translational modifications of *SOX2* are shown to regulate its protein stability and transcriptional activity. Akt-mediated phosphorylation at Thr118 promotes the transcriptional activity of *SOX2* in ESCs (Burchfield et al., 2015). Furthermore, Mallaney et al., (2019) showed that Set7 methylates *SOX2* at K119, inhibiting Sox2 transcriptional activity and inducing Sox2 ubiquitination and degradation. Our study found that KDM6B occupies the SOX2 promoter in matrix-detached conditions, probably reduces the repressive H3K27me3 from its promoter, and increases its expression. Previous research by Fang et al., (2014) had shown that GSK J4 treatment reduces the expression of stemness genes, i.e., SOX2, Nanog, and OCT4 in breast cancer stem cells; however, they did not present direct evidence that KDM6B occupies the promoter of these genes in cancer cells.

CD44 is a prominent and well-established marker of stem cells, and its expression can be regulated at epigenetic levels. DNA methylation of the CD44 promoter by DNA methyltransferases (DNMTs), MBD1, MBD2, and MeCP2 is well-reported (Eberth et al., 2010; Müller et al., 2010). Our work found that KDM6B occupies the promoter of CD44 during matrix detachment of cancer cells. Our finding of KDM6B-mediated transcriptional regulation of CD44 is in apparent agreement with (Eberth et al., 2010; Li et al., 2014; Itoh et al., 2019), who showed *CD44* as a bonafide target of KDM6B in immune and leukemic cells. However, we firmly believe this is the first report to show epigenetic regulation of *CD44* by KDM6B in various concrete cancer types. Furthermore, a positive correlation of the gene expression from two different metastatic cancer data suggesting the involvement of KDM6B in regulating HIF1α, *SOX2*, and *CD44* further indicates the stemness regulatory function of KDM6B in various cancer types.

Hypoxia has been recently associated with the matrix detachment of cancer cells and can regulate the matrix-detached cancer cells (Mohammed and Razeeth, 2021). Our results are in explicit agreement with this study as we also found a clear induction in the expression of HIF and its target genes. . We found that KDM6B occupies the promoter HIF1α and regulates its expression in matrix-detached cancer cells Hypoxia regulates global transcription in multiple ways, and regulation of the expression and activity of histone demethylases is one of them (Heinemann et al., 2014). Therefore, we target to explore the expression pattern of hypoxia-regulated histone demethylases in matrix-detached cancer cells. Our study showed significant and consistent upregulation of KDM6B in all cell lines at the time points tested. Thus, KDM6B plays a crucial and dual role in cancer initiation and progression through binding to promoters of oncogenes or suppressor genes. Furthermore, KDM6B was associated with aggressiveness and enhanced migratory properties of various cancer types (Burchfield et al., 2015; Fu et al., 2019).

## Conclusion

Our data suggest that the rapid and sustained upregulation of KDM6B following matrix detachment is necessary for *SOX2* and *CD44*-mediated stemness to enhance anchorage-independent survival of various cancer patients’ cells. Additional consequences of the context-specific increase and regulation of HIF1α by KDM6B might be likely to further aid in survival in response to changing nutrient microenvironments. Our study highlights an essential role of KDM6B in cancer and has important implications for targeting this protein for anticancer therapies.

## Data Availability

The original contributions presented in the study are included in the article/Supplementary Material; further inquiries can be directed to the corresponding author.
