# Supplementary figures and images for: The Histone H3K27me3 Demethylases KDM6A/B Resist Anoikis and Transcriptionally Regulate Stemness-Related Genes
Source: Front Cell Dev Biol. 2022 Feb 2;10:780176. doi: 10.3389/fcell.2022.780176 (PMC8847600; doi:10.3389/fcell.2022.780176)

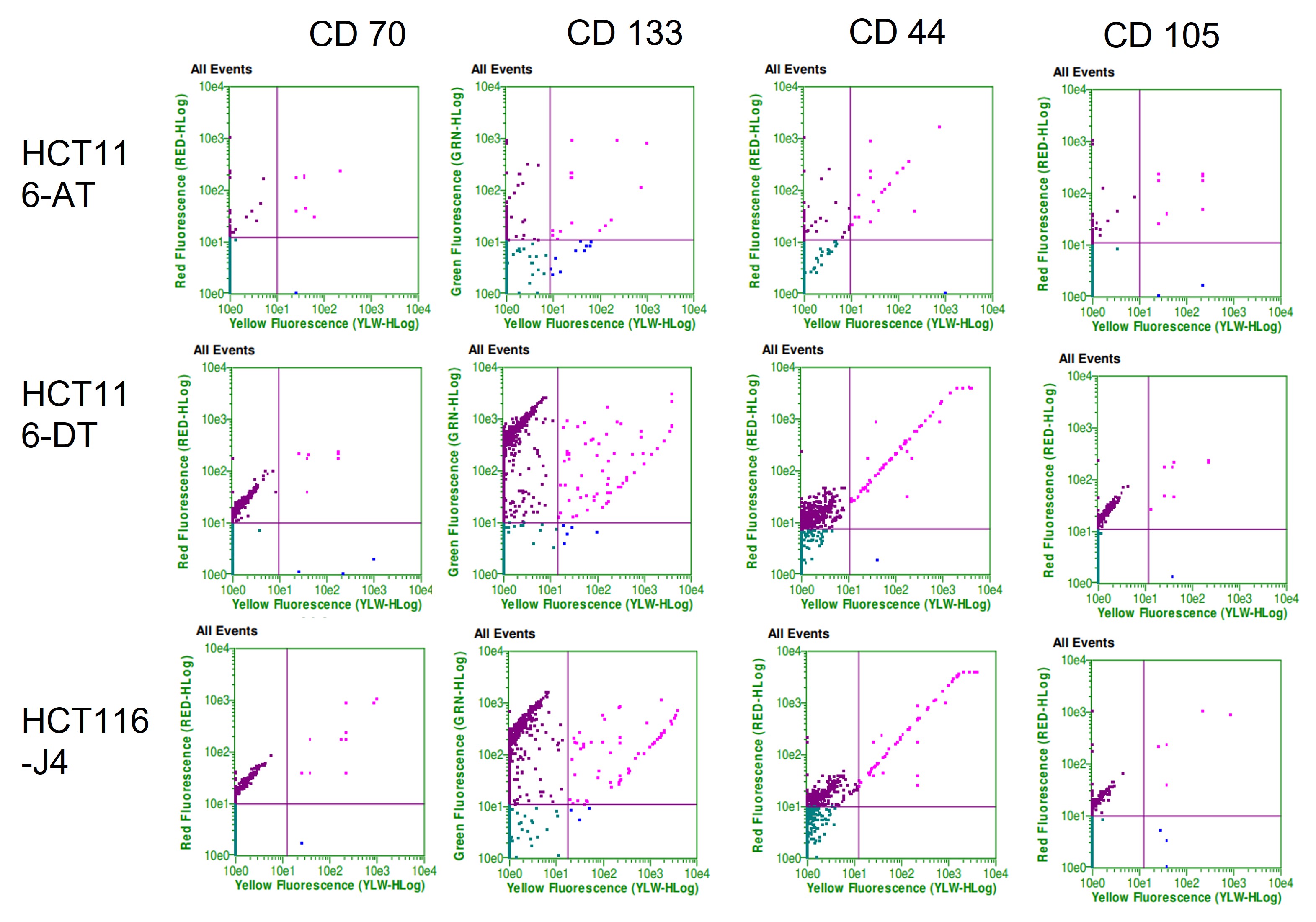

Supplement: Supplementary file 1 [file Image3.JPEG]

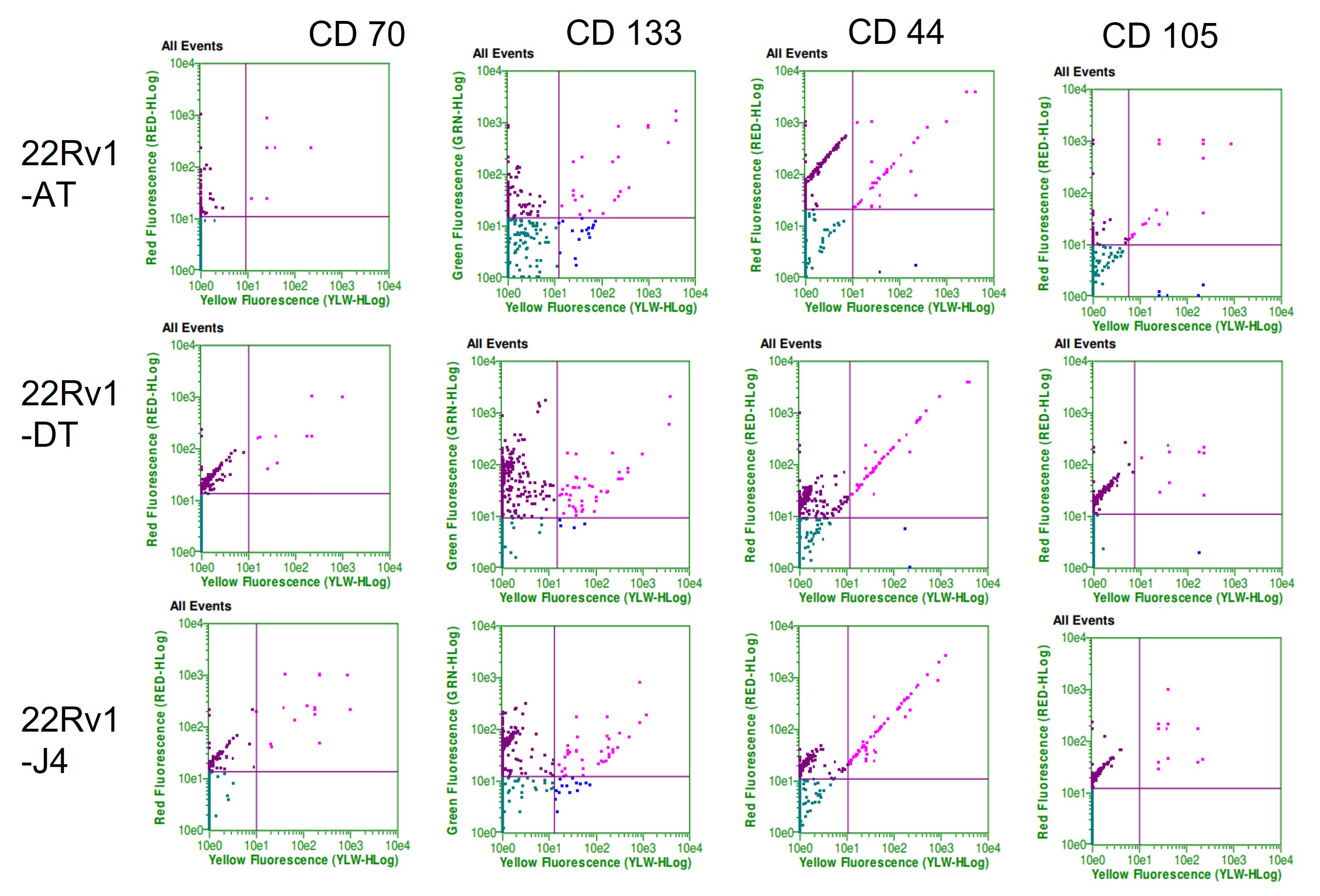

Supplement: Supplementary file 2 [file Image4.JPEG]

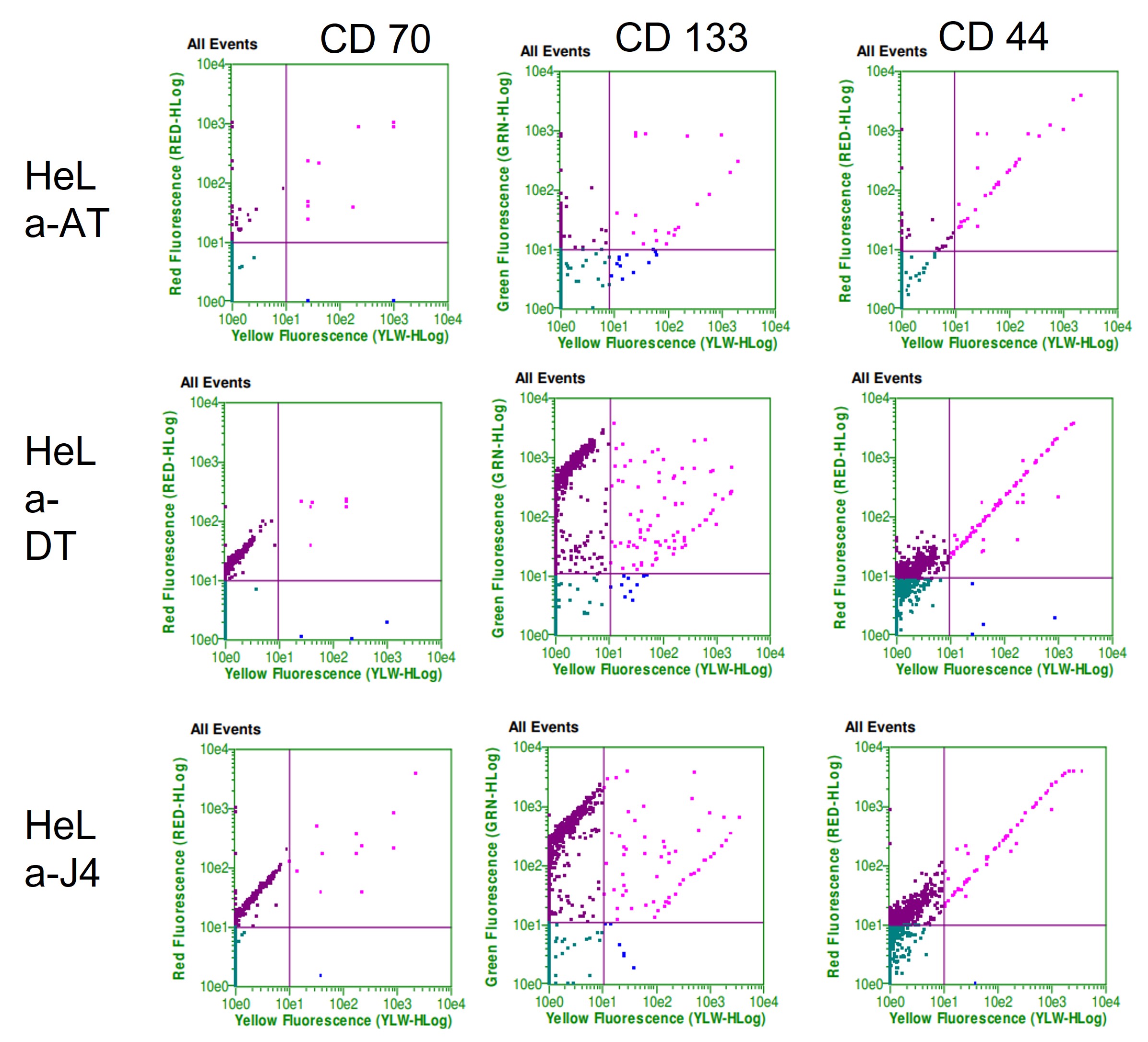

Supplement: Supplementary file 3 [file Image2.JPEG]

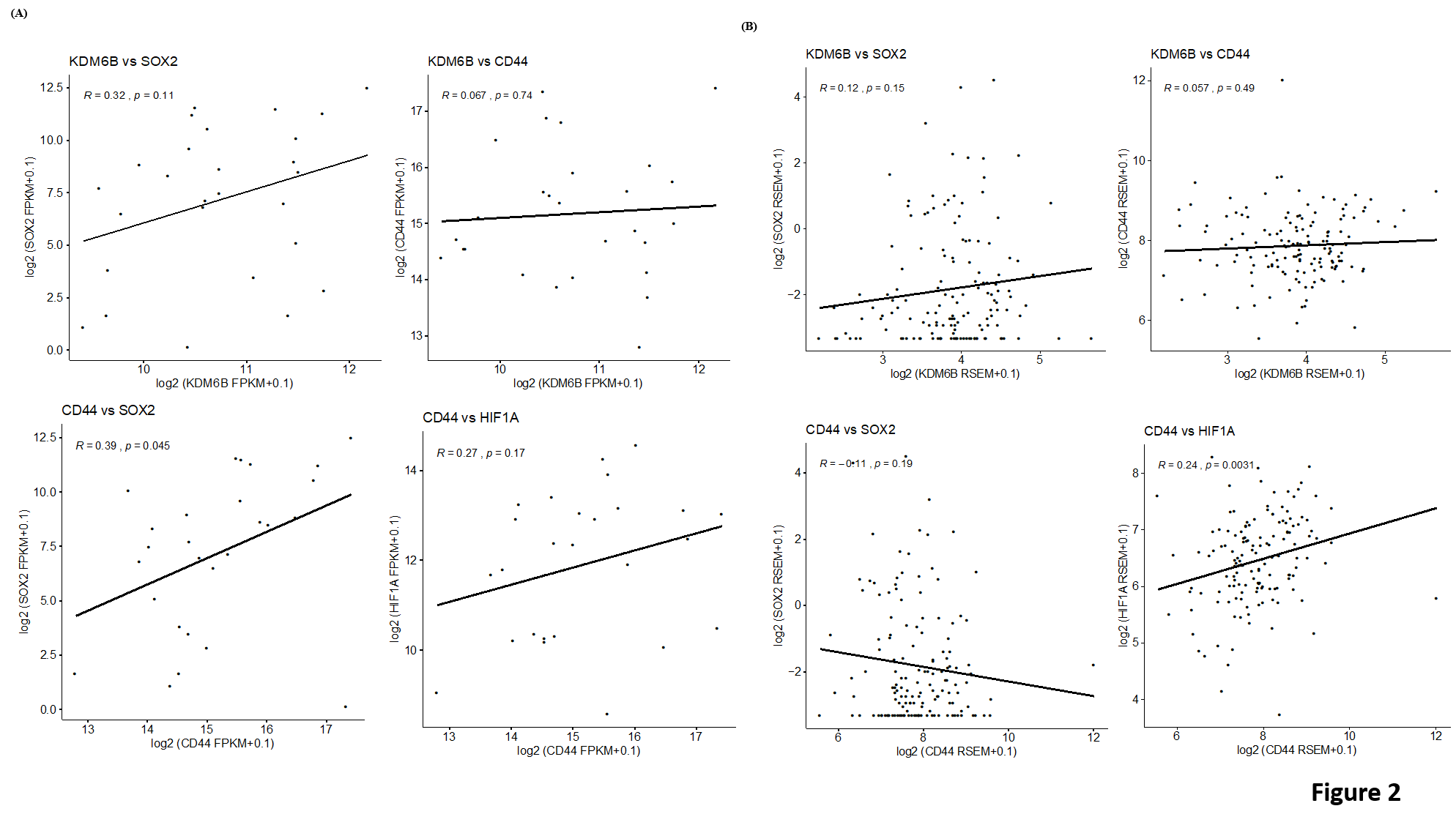

Supplement: Supplementary file 4 [file Image1.TIF]
